# Increased Potency of a Bi-specific TL1A-ADAM17 (TACE) Inhibitor by Cell Surface Targeting

**DOI:** 10.3389/fmolb.2017.00061

**Published:** 2017-08-22

**Authors:** Tomer Weizman, Itay Levin, Marianna Zaretsky, Irit Sagi, Amir Aharoni

**Affiliations:** ^1^Department of Life Sciences, Ben-Gurion University of the Negev, Beersheba Beersheba, Israel; ^2^The National Institute for Biotechnology in the Negev, Ben-Gurion University of the Negev, Beersheba Beersheba, Israel; ^3^Department of Biological Regulation, Weizmann Institute of Science Rehovot, Israel

**Keywords:** bi-specific inhibitors, protein engineering, pro-inflammatory cytokines, metalloproteases, TL1A, TACE, ADAM17

## Abstract

Inflammatory bowel disease (IBD) is a multifactorial disease characterized by the dysregulated activity of many pro-inflammatory factors. Thus, bi-specific inhibitors for the simultaneous inhibition of two pro-inflammatory factors can exhibit high therapeutic potential. Here, we developed a novel bi-specific inhibitor targeting the TL1A cytokine and ADAM17/TACE metalloprotease. Biochemical analysis of the bi-specific inhibitor revealed high TL1A binding and TACE inhibition that is similar to the two respective mono-specific inhibitors. Interestingly, cell based assays for TL1A inhibition revealed strong synergism between the inhibitory domains showing an up to 80-fold increase in potency of the bi-specific inhibitor. The dramatic increase in potency is associated with binding to cell membranes through the TACE inhibitory domain leading to increased concentration of the inhibitor on the cell surface. Our study highlights the high potential of the simultaneous targeting of cell surface metalloprotease (TACE) and soluble pro-inflammatory cytokine (TL1A) as a potential therapeutic approach in IBD.

## Introduction

Inflammatory bowel disease (IBD) is the progressive, relapsing, chronic inflammation of the digestive tract, which primarily includes ulcerative colitis and Crohn's disease. This debilitating disease is steadily becoming a worldwide medical concern, with increasing prevalence in industrialized and developing countries (Molodecky et al., 2012). Generally, IBD is characterized by a dysregulated immune response leading to tissue damage in the gastrointestinal tract (Abraham and Cho, 2009; Khor et al., 2011). To date, sporadic reports suggest a link between extracellular matrix (ECM) remodeling and immune response during intestinal inflammation with little to no mechanistic information (Shimshoni et al., 2015). The mutltifactorial nature of IBD where multiple immune modulators, ECM remodeling enzymes and other factors affect disease progression calls for the development of inhibitors that can simultaneously target two pro-inflammatory pathway leading to increased potency and reveal a possible cross-talk between these pathways.

TNF-like ligand 1A (TL1A) is a newly described member of the TNF superfamily that has been shown to be involved in promoting a range of autoimmune diseases including IBD, rheumatoid arthritis (RA), and asthma through the generation and activation of the type-1 T helper cell (T_H_1) and type-17 T helper cell (T_H_17; Bamias et al., 2003, 2006; Bull et al., 2008; Fang et al., 2008). TL1A was shown to be expressed by endothelial cells, lymphocytes, and monocytes and its expression is enhanced in the intestinal tissues of patients with IBD (Bamias et al., 2003; Cassatella et al., 2007). TL1A is currently the only known ligand for death receptor 3 (DR3), which is predominantly expressed by activated T cells and endothelial cells (Chew et al., 2002; Bamias et al., 2003). The binding of TL1A to DR3 was shown to boost the secretion of IFN-γ from T cells by acting in synergy with IL-12 and IL-18 and thus direct the immune response toward a T_H_1 like response (Papadakis et al., 2004; Cassatella et al., 2007). To inhibit TL1A, we have recently utilized directed engineered to generate an improved soluble DR3 “trap” receptor (Levin et al., 2017). These DR3 extracellular domain (ECD) variants, consisting of 171 residues, exhibit high stability, TL1A binding affinity and potent inhibition of TL1A induced cytokine secretion from T cells.

Dysregulated activity of A Disintegrin And Metalloproteinase 17 (ADAM17)/TNFα Converting Enzyme (TACE) is associated with inflammatory disorders, cancer progression, and neurodegenerative disease by releasing regulatory membrane-tethered proteins like TNFα, IL6R and EGFR ligands (Arribas and Esselens, 2009; Gooz et al., 2009; Scheller et al., 2011; Rose-John, 2013). Although, specific inhibition of TACE is thought to be a viable strategy for inflammatory disorders and malignancies treatment, the generation of effective inhibitors *in vivo* was proven challenging (Wilson et al., 2007; Devel et al., 2010). Recently, we have generated a stable form of the auto-inhibitory TACE prodomain (pTACE), which specifically inhibit cell-surface TACE, but not the related ADAM10 (Wong et al., 2016). We found that TPD is a potent, highly selective and efficacious *in vivo* modulator of both human and mouse TACE sheddase activity. Thus, we have shown that harnessing an endogenous inhibitory mechanism for reconstitution of the TACE zymogen *via* exogenous addition of its natively folded prodomain is a promising approach for protein-based inhibitor design. We showed that pTACE significantly attenuated TACE-mediated disease models of sepsis, rheumatoid arthritis (RA) and IBD.

In recent years, the generation of bi-specific biological reagents that can simultaneously target two factors focused on bi-specific antibodies (Spiess et al., 2013; Smith et al., 2015). Bispecific antibodies were developed in a variety of different formats, however, with many technical difficulties due to their complex nature. Single chain variable region (scFv) modules were employed to force the assembly of binding components into the desired configuration (Todorovska et al., 2001). Concerns with many of these formats include a tendency to aggregate, difficulties in production, short serum half-lives, or potential of immunogenicity. In addition, strategies for the generation of native antibody where the heavy chain Fc-Fc interface is engineered with “knobs” and “holes” or electrostatic charges to actively promote the formation of the desired heterodimers were developed (Ridgway et al., 1996).

Here, we combined two engineered protein domains, an engineered sDR3 variant and pTACE, to generate a bi-specific inhibitor that can simultaneously inhibit TL1A and TACE. To increase the stability and activity of the domains, the construct was fused to an Fc region of the human IgG_1_ at the C-terminal. We found that the bi-specific inhibitor exhibits similar biochemical activity including TL1A binding and TACE inhibition as the respective non-fused mono-specific inhibitors. Interestingly, the bi-specific inhibitor demonstrated strong synergistic effect between the domains with an increase of up to ~80-fold in inhibiting TL1A induced cytokine secretion or apoptosis in T cells or TF-1 cell line, respectively. We found that this strong synergistic effect is associated with a significant increase in binding of the bi-specific inhibitor to the TF-1 cell membrane through the pTACE domain, leading to increased local soluble DR3 concentration on the cell membrane. Thus, our data suggests that bi-specific inhibitors can exert high potency through cell surface targeting in gut cell population expressing both TACE and DR3.

## Materials and methods

### Generation of plasmids

**Figure 1:**
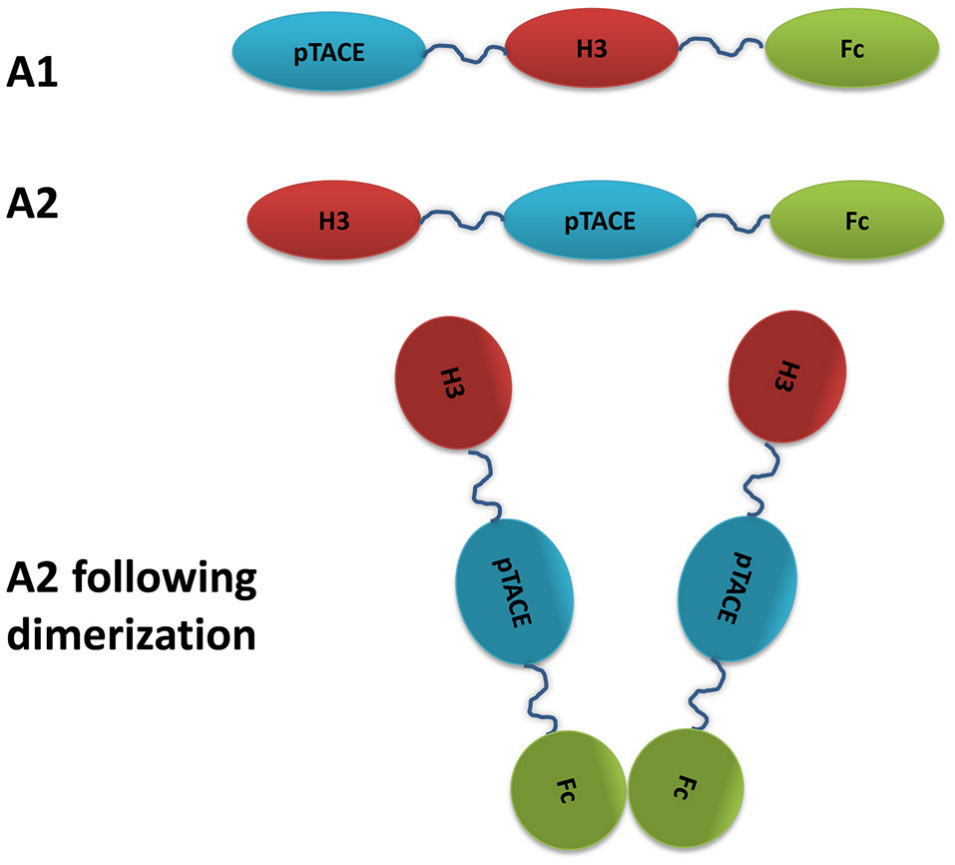
Schematic representation of the bi-specific soluble DR3 and pTACE inhibitor targeting TL1A and TACE. H3 is an engineered variant of soluble DR3 exhibiting high affinity and stability relative to the WT (Levin et al., 2017).

The *E. coli* E. Cloni strain (Lucigen) was used for cloning and plasmid extraction. The A1 and A2 inhibitors (Figure 1) were constructed by PCR amplification from ProTACE3mut (Wong et al., 2015) and pFUSE-DR3 (H3 variant) (Levin et al., 2017). The resulting fragments were purified and assembled by a secondary PCR to form H3-linker-6xHis-proTACE (A2) and proTACE-linker-6xHis-H3 (A1). The assembled proTACE and H3 fragments were digested and cloned into pFUSE-hIgG1e3-Fc (Invivogen) with *NcoI* and *BglII* to yield an open reading frame coding for a signal peptide followed by H3-linker-6xHis-TACE-Fc (A2) and TACE-linker-6xHis-H3-Fc (A1).

### Cell culture

H293F were grown in Freestyle media with 1x Pen/strep solution (Biological Industries, Beit-Haemek, Israel), at 37°C, 8% CO_2_ with shaking. PBL were grown at a concentration of 1^*^10^6^/ml in RPMI 1640, with 10% FBS 2 mM Glutamine, 1x Pen/strep solution supplemented with 10% heat inactivated FBS. TF-1 Cells (ATCC–CRL2003) were grown in RPMI supplemented with 10% FBS, 2 mM Glutamine, 2 mM GM-CSF (Peprotech), 1 x Pen/step solution (all ingredients for cell culture and antibiotics were obtained from Biological Industries, Beit-Haemek, Israel). The culture was maintained between 3^*^10^4^ and 5^*^10^5^ viable cells/ml.

### Large scale protein expression and purification

#### Purification of proTACE

pMALC2 containing proTACE3mut was transformed into *E. coli* Rosetta strain (Novagen) and grown over night on LB agar with 100 μg/ml ampicillin and 30 μg/ml chloramphenicol. One colony was used to inoculate a 10 ml LB and then transferred into 200 ml LB 100 μg/ml ampicillin and 30 μg/ml chloramphenicol the culture was allowed to grow till OD_600_ of 0.6 and then IPTG was added to a final concentration of 0.4 mM, and the culture was grown at 15°C, 250 rpm overnight. The cells were centrifuged at 3220 rcf for 15 min and 100 ml lysis buffer containing 50 mM TRIS pH 8.0, 300 mM sodium chloride, 20 mM imidazole, and 0.05 mg/ml lysozyme, 1250 U benzonaze, 20 μl protease Inhibitor cocktail II (CalBioChem) was added for 30 min incubation on ice. The lysate was then sonicated and centrifuged at 10,000 rcf, 4°C for 1 h. The supernatant was then removed and filtered through 0.45 micron filter (Millipore) and the extract was loaded into a column containing Ni-NTA Agarose resin (Thermo scientific) that was pre-equilibrated with wash buffer containing 50 mM Tris pH 8.0, 300 mM sodium chloride, and 20 mM imidazole. Subsequently the column was washed with 4 column volume (CV) of wash buffer and the sample was eluted and fractionated by applying 2 CV of 50 mM Tris pH 8.0, 300 mM sodium chloride, and 250 mM imidazole. The eluted sample was analyzed by SDS-PAGE, dialysed twice against 50 mM Tris at pH 8.0 at 4°C and flash freezed in liquid nitrogen and stored in −80°C.

### Purification of A1 and A2

HEK293F cells were grown in Freestyle serum free media (Invitrogen) and were transiently transfected using Genetran III (Biomiga) as recommended by the manufacturer and the transfected cells were incubated with shaking at 37°C at 8% CO_2_ incubator. Next, 7 days post transfection the cell media was collected, filtered and loaded into a Ni-NTA Agarose resin (Thrmo scientific) column pre-equilibrated with 50 mM Tris pH 8.0, 300 mM NaCl, and 20 mM imidazole. The column was then washed with 20 CV of 50 mM Tris pH 8.0, 300 mM NaCl, 20 mM imidazole. The protein was eluted with 3 CV of 50 mM Tris pH 8.0, 300 mM NaCl, 500 mM imidazole. The eluted fractions were analyzed by SDS-PAGE, and selected fractions were dialyzed twice against 50 mM Tris pH 8.0, flash freezed in liquid nitrogen and stored at −80°C.

### Western blot analysis

Cell culture media was collected 7 days post transfection, run on 10% Tris gel and transferred to a PVDF membrane. The membrane was blocked with PBS containing 3% skim milk (Sigma) washed three times with PBS containing 0.05% Tween-20 (Sigma) (PBST) and incubated over night with 1.3 μg/ml Goat anti Human IgG (Jackson Immunoresearch) or 0.1 μg/ml Anti DR3 Biotin (R&D biosystems). Subsequently the membrane was washed three times with PBST and incubated for 1 h with 0.08 μg/ml Horse Reddish Peroxidase (HRP) conjugated Donkey anti-Goat or 0.1 μg/ml HRP conjugated streptavidin (Jackson Immunoresearch). The membrane was developed with EZ-ECL (Biological industries, Israel) and analyzed using FusionFX (Vilber Lourmat).

### ELISA for monitoring TL1A-DR3 interaction

ELISA plates (Griener Microlon 96W) were incubated with 100 μl of 0.66 μg/ml monoclonal mouse α-TL1A antibodies (Santa Cruz) for 1 h, washed with PBST and 100 μl of 1 μg/ml TL1A (peprotech), were added to the plate for an additional hour. The plates were then washed with PBST and blocked by incubation with 150 μl of PBS supplemented with 3% skim milk for 1 h. Following blocking, the plates were washed and incubated with 100 μl of media of HEK293T transfected with H3 or A2 variants harvested 72 h post transfection, ELISA plates were shaken for additional hour. DR3-Fc (R&D Systems) was added at a concentration of 2 μg/ml as a positive control, and PBS supplemented with 1% BSA served as a negative control. Plates were then washed with PBST, incubated with 100 μl of 0.05 μg/ml of biotinylated goat polyclonal α-DR3 antibodies (R&D Systems), followed by incubation with secondary peroxidase-conjugated streptavidin (Jackson, 1:10,000 dilution). Finally, 100 μl of horseradish peroxidase (HRP) chromogenic 3,3′,5,5′-tetramethylbenzidine (TMB) substrate solution (Dako) were added. The reaction was stopped by the addition of 1 M sulfuric acid and recorded at 450 nm using a Tecan Infinite M200 plate reader.

### TACE *in-vitro* activity assay

Recombinant human ADAM17/TACE (R&D) was diluted as recommended by the manufacturer to a final concentration of 2.85 nM and the A2 or proTACE were added in various concentrations, the mixture was incubated for 5 min at RT. Then 10 μM fluorogenic Peptide Substrate Mca-PLAQAV-Dpa-RSSSR-NH2 (R&D systems) was added, and cleavage of the fluorogenic substrate from the Dpa quencher was monitored in Tecan infinite M200 plate reader with excitation set at 320 nm and emission set at 405 nm.

### Mouse peritoneal macrophages cell based assay of TNF-α secretion inhibition

Mouse peritoneal macrophage cells were harvest, 4 days post Thioglycollate injection, and incubated overnight in RPMI 1640, with 10% FBS 2 mM Glutamine, 1x Pen/strep solution supplemented with 10% heat inactivated FBS (both from Biological Industries, Beit-Haemek, Israel). Macrophages medium was replaced and 2.5 ng/ml LPS and 1 μM A2 or proTACE were added and incubated for 3 h at 37°C. The macrophages medium was collected and the TNF-α in the medium was determined using mouse TNF-α DueSet ELISA kit (R&D systems) as recommended by the manufacturer.

### PBL cell based assay for the inhibition of TL1A-induced IFN-γ secretion

PBMC were isolated from blood of normal healthy volunteers using Histopaque®-1077 (Sigma) according to the manufactures instructions. The PBL fraction was isolated following incubation of the PBMC in complete RPMI in a flask at 37°C for overnight and the non-adherent fraction was designated as PBL. Blood from normal healthy volunteers was obtained from the blood bank of Israel under an agreement with Ben-Gurion University (BGU) in which only PBMC/CD4 cells will be produced from the blood donations. All procedures for isolating the PBMC/CD4 cells were performed with approved protocols according to the regulation and safety of BGU. For determining soluble DR3 inhibition of TL1A induced IFN-γ secretion, PBL cells were incubated in RPMI containing 10% FBS with IL-12 (2 ng/ml), IL-18 (50 ng/ml), TL1A (200 ng/ml), and A2 or H3 at different concentrations for 48 h. The cultured media was collected and the levels of IFN-γ were quantitated using a commercial ELISA kits (PeproTech) according to manufacturer description.

### TF-1 cell based assay for the inhibition of TL1A-induced apoptosis

TF-1 cells were seeded at 75,000 cells/well (7.5 × 10^5^/ml) in RPMI medium containing 1% FBS (Biological Industries, Beit-Haemek, Israel), in a black 96-well plate with clear bottom (Greiner bio-one) at a final volume of 100 μl. Next, 100 ng/ml TL1A, 10 μg/ml cycloheximide and A2, H3 or proTACE were added. After 6 h 100 μl of lysis buffer (50 mM HEPES pH 7.35, 1 mM EDTA, 1% NP-40 detergent, 25 μM DEVD-AMC) was added to each well, and cleavage of DEVD-AMC by caspase-3 was monitored for 60 min in a Tecan Infinite M200 plate reader with excitation and emission set at 350 nm and 450 nm, respectively.

### Flow cytometer binding assay of A2 to TF-1 cells

TF-1 cells (1.5^*^10^5^) in a volume of 150 μl were incubated with the indicated concentrations of A2, H3 or pTACE in RPMI 1% FBS for 1 h at 37°C. Next, the cell were centrifuged at 200 rcf for 5 min at 4°C and washed three times with 200 μl PBS, 1% BSA. The washed cells were re-suspended in 50 μl PBS containing 1% BSA and goat anti-human IgG Allophycocyanin (APC) conjugate (Jackson immuneresearch) was added to a final concentration of 10 μg/ml. The cells were incubated for 30 min at RT and then washed three times with PBS containing 1% BSA. The cells where resuspended in 500 μl of PBS containing 1% BSA and analyzed on Acuri C6 flow cytometer (BD Biosciences).

### Statistical analysis

All experiments performed with mouse peritoneal macrophages and TF-1 cells were repeated at least three times. Data values are presented as means of the three experimental repeats with standard deviation presented as error bars. To examine significance differences between the inhibition level of TNF-α release from macrophages upon different treatments, the data was analyzed by unpaired student *t*-test. Statistically significance differences were denoted with a star and only *p*-value below 0.05 were considered as statistically significant.

## Results

### Generation of bi-specific DR3-pTACE inhibitor

For the generation of bi-specific DR3-pTACE inhibitor, we utilized an engineered DR3 variant, termed H3, which exhibits improved TL1A binding affinity and higher stability relative to the WT soluble DR3 receptor. In addition, the H3 variant showed higher inhibition of TL1A induced IFN-γ secretion and apoptosis in CD4^+^ and TF-1 cells, respectively, relative to the WT receptor (Levin et al., 2017). For TACE inhibition, we used a previously described pTACE domain containing mutations at the natural furin cleavage site to maintain the integrity of our bi-specific inhibitor (Wong et al., 2015). The pTACE domain, expressed and purified from *E. coli*, was previously shown to exhibit significant inhibition of TACE both *in vitro* and on cell membrane (Wong et al., 2016). Our bi-specific inhibitor consists of three major domains: the pTACE and H3 that specifically bind TACE and TL1A, respectively, and an Fc domain derived from human IgG1. The role of the Fc domain is to extend the serum half-life of the inhibitor and to promote dimerization of the bi-specific proTACE-H3 inhibitor (Wu and Sun, 2014). To test different constructs for optimizing protein expression in mammalian cell line, we have generated two bi-specific inhibitors consistent of pTACE-H3-Fc and H3-pTACE-Fc (termed A1 and A2, respectively, Figure 1). Both constructs contain a 16 residue flexible linker containing a six histidine tag between the pTACE-H3 and H3-pTACE domains to facilitate protein purification. These constructs were cloned into a mammalian expression vector containing a signal peptide to facilitate the secretion of the inhibitor into the growth media.

To facilitate protein expression and purification, the A1 and A2 bi-specific inhibitors were transiently transfected and expressed in HEK293F cells in serum free media for 7 days (see Section Materials and Methods for details). Using western blot analysis we found that the H3-pTACE-Fc (A2) is expressed and secreted much better than the pTACE-H3-Fc (A1) (Figure S1A). The presence of the H3 domain was further verified by western blot analysis with polyclonal antibodies against the DR3 domain of the protein (Figure S1B). To test whether the A2 is secreted as an active protein, the supernatant of HEK293F containing the A2 protein was subjected to a TL1A ELISA binding assay (see Section Materials and Methods for details). We found that the secreted A2 exhibits high TL1A binding signal that is dose dependent (Figure S2). Next, the A2 was purified on Ni-NTA beads and the pure A2 migrated on an SDS-PAGE gel as a band of ~100 kDa, higher than the calculated 70 kDa, suggesting that protein undergo significant posttranslational modifications (Figure S1C).

### Biochemical analysis of the bi-specific A2 inhibitor

**Figure 2:**
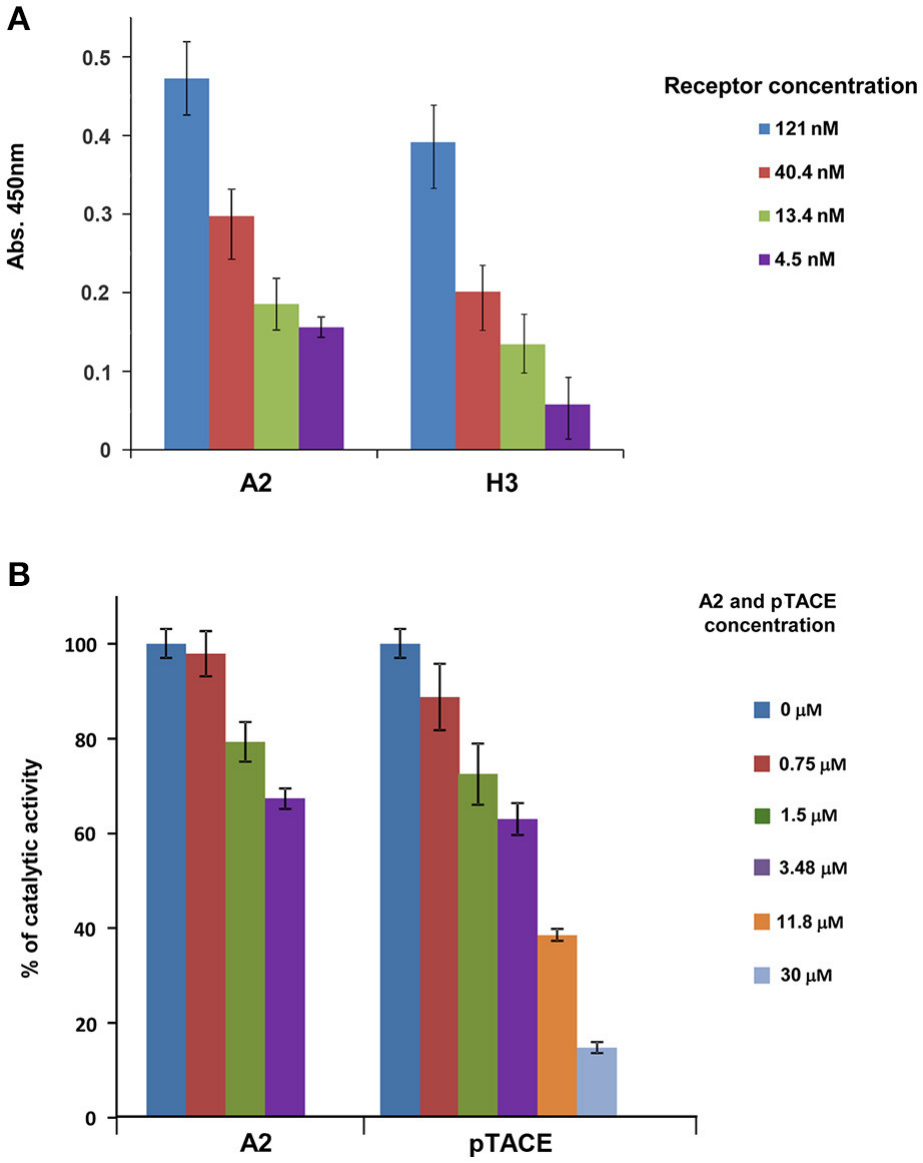
The A2 bi-specific inhibitor exhibits similar biochemical activity as the two mono-specific H3 (DR3) and pTACE inhibitors. **(A)** Binding of A2 to TL1A in comparison to the mono-specific H3 protein. Binding analysis was performed by ELISA using plates that were pre-coated with TL1A (see Section Materials and Methods for detailed description). **(B)** Inhibition of TACE by A2 in comparison to the mono-specific pTACE inhibitor. Inhibition of TACE activity was assessed using a fluorogenic peptide substrate DEVD-AMC.

To examine whether the bi-specific inhibitor A2 containing the H3 and pTACE domains is fully functional, we examined the biochemical activity of the two inhibitory domains relative to the respective mono-specific inhibitors. We first analyzed the binding of the H3 domain in A2 to TL1A using ELISA relative to the purified mono-specific H3 variant. We found that the pure bi-specific A2 and H3 proteins exhibit similar dose dependent binding to immobilized TL1A suggesting that A2 maintains full TL1A binding activity relative to H3 (Figure 2A). To test the ability of A2 pTACE domain to inhibit TACE proteolytic activity, we first established an activity assay for TACE using a fluorogenic peptide substrate derived from TACE cleavage site in TNFα. Next, we added increased A2 concentrations to TACE and the reduction in enzymatic proteolytic activity toward the peptide substrate was measured and compared to the bacterial pTACE mono-specific inhibitor (Wong et al., 2016). We found that A2 and pTACE exhibit similar TACE inhibition up to a concentration of 3.5 μM of inhibitor (Figure 2B). Full inhibition with the bacterial pTACE was observed at a concentration of 30 μM, a concentration that was beyond the production capacity of our mammalian cell expression system. Overall, these results demonstrate that the bi-specific A2 inhibitor maintains full activity capable of efficient TL1A binding and TACE inhibition.

### Cell based assays for the bi-specific A2 inhibitor

**Figure 3:**
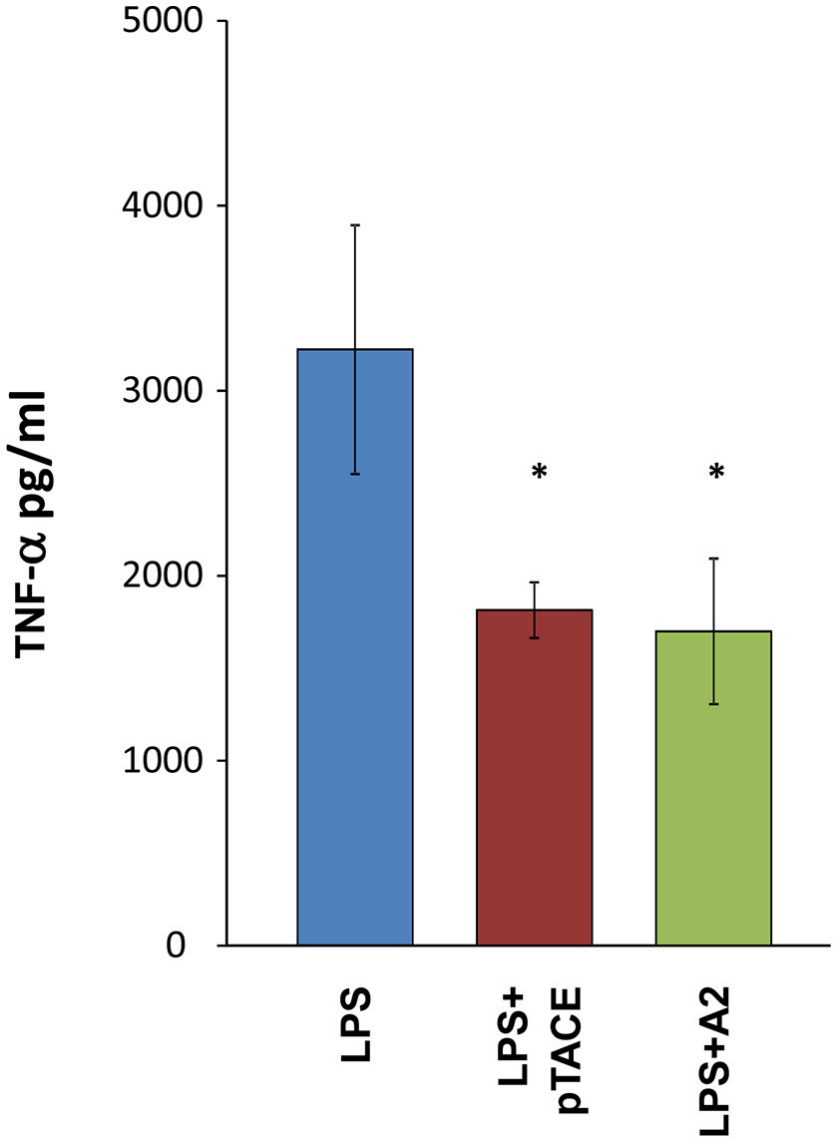
Similar inhibition level of TNF-α release from macrophages following incubation with A2 and the mono-specific pTACE inhibitor. The levels of TNF-α secretion from mouse peritoneal macrophages were determined following 3 h of LPS stimulation at a concentration of 2.5 ng/ml with or without 1 μM of A2 or pTACE. Media supernatant was analyzed by ELISA for detection of TNF-α levels, the TNF-α values were calculated according to TNF-α calibration curve, ^*^P < 0.05.

**Figure 4:**
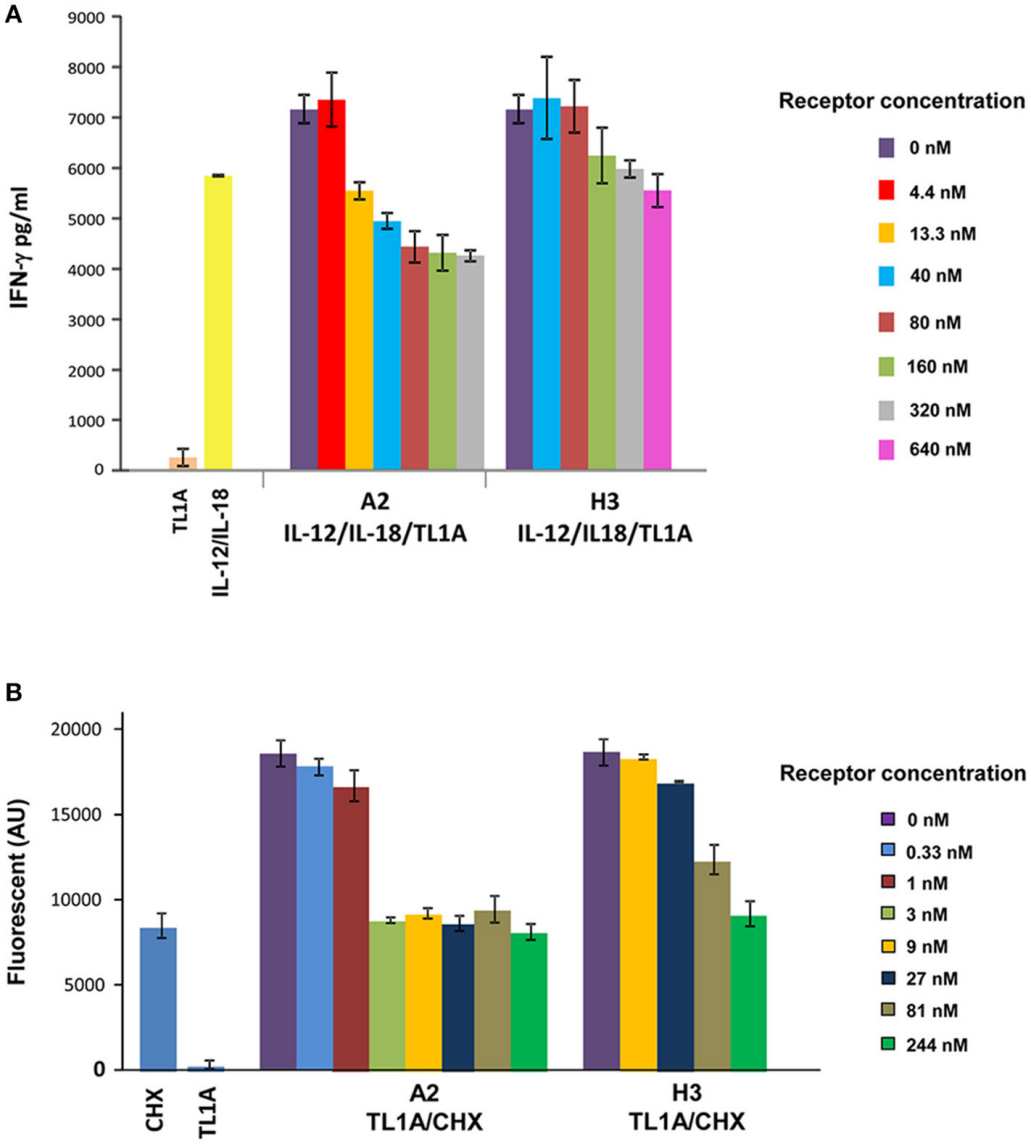
Increased potency of A2 vs. H3 (DR3) in inhibiting TL1A induced IFN-γ secretion and apoptosis in PBL and TF-1 cell line, respectively. **(A)** Inhibition of TL1A-induced secretion of IFN-γ in human PBL by increased concentration of A2 and H3. Cells were incubated for 72 h with 200 ng/ml TL1A, 20 ng/ml IL-12 and 50 ng/ml IL-18, and different concentrations of A2 and H3 inhibitors. The 1:10 diluted cell supernatant was analyzed by ELISA for detection of IFN-γ levels. The IFN-γ values were calculated according to IFN-γ calibration curve. **(B)** Inhibition of TL1A-induced apoptosis in TF-1 cells by increased concentration of A2 and H3. Cells were incubated for 6 h with 8 μg/ml of cyclohexamide (CHX) and 75 ng/ml of TL1A and the indicated concentration of A2 and H3 receptors. Following incubation, lysis buffer containing the caspase-3 fluorescent substrate DEVD-AMC was added and enzyme activity was monitored for 10 min. The data presented in the PBL and TF-1 experiments is the average of three independent repeats of each experiment and the error bars represent the standard deviation from the average.

*In vivo* TACE activity involves proteolytic shedding of pro-inflammatory cytokines and growth factors located on the cell membrane (Chalaris et al., 2010). Previously, macrophages were shown to respond to LPS by activating TACE cleavage of membrane bound proTNF-α leading to the release of soluble TNF-α (Bell et al., 2007). To examine the ability of A2 to inhibit TACE TNF-α “shedase” activity, we tested the levels of TNF-α released from mouse peritoneal macrophages in the absence or presence of A2 and pTACE inhibitors. We found that A2 and pTACE exhibit similar reduction in the level of TNF-α released to the medium compared to control cells (Figure 3). Next, we examined the ability of A2 to inhibit TL1A induced cytokine secretion from immune cells. Previously, it was shown that TL1A cooperate with IL-12 and IL-18 to induce IFN-γ secretion from peripheral blood lymphocytes (PBL; Papadakis et al., 2004). We utilized this cell based assay to examine the ability of A2 and H3 variants to inhibit TL1A induced IFN-γ secretion by competing with the endogenous DR3 cell surface receptor. Interestingly, we found that that A2 completely inhibits IFN-g secretion at 13.3 nM the H3 reaches a similar level of inhibition at only 640 nM reflecting ~50-fold higher potency in inhibiting TL1A induced IFN-γ secretion from PBL relative to H3 mono-specific inhibitor (Figure 4A). These results indicate a strong synergism between H3 and the pTACE domains in A2 in inhibiting TL1A induced IFN-γ secretion from PBL.

To further examine the synergism between H3 and pTACE in inhibiting TL1A, we employed a second cell based assay. TL1A was previously shown to significantly enhance cyclohexamide (CHX) induced apoptosis in TF-1 cells detected by the increase in caspase-3 activity toward the DEVD-AMC fluorescent substrate (Migone et al., 2002). Previously, we utilized this assay to demonstrate that the addition of increasing concentrations of H3 led to a potent inhibition of TL1A induced apoptosis in TF-1 cells (Levin et al., 2017). Here, we utilized this cell based assay to compare the potency of A2 and H3 in inhibiting TL1A induced apoptosis of TF1 cells. We found that A2 inhibits TL1A induced apoptosis at a significantly lower concentration than the H3 mono-specific inhibitor (Figure 4B). Specifically, we found that while H3 completely inhibits TL1A induced apoptosis at a concentration 244 nM, the A2 led to a complete inhibition at a concentration of 3 nM reflecting a more than 80-fold increase in potency (Figure 4B).

### The synergism between H3 and pTACE is associated with binding to cell membrane

The increased potency of the bi-specific A2 inhibitor relative to the H3 indicates a strong synergism between the H3 and the pTACE domains in A2. One possible mechanism for such synergism is the independent activity of the pTACE domain in preventing apoptosis of the TF-1 cells. Previously, TACE was reported to be involved in inducing apoptosis in germ cells and neutrophils and its chemical inhibition resulted in reduced apoptosis (Wang et al., 2011; Lizama et al., 2012). To test this hypothesis, we examined the levels of TL1A induced apoptosis following cell incubation with a wide range of the mono-specific pTACE concentrations. We found that pTACE did not lead to the inhibition of TL1A induced TF-1 apoptosis under the assay conditions suggesting that the high potency of A2 is not due to direct TACE inhibition (Figure S3). To examine whether the improved potency of A2 is due to fusion of the H3 to the pTACE domain, we examined the potency of A2, H3, and the combination of H3 and pTACE domains at different inhibitor concentrations. We found that the combination of pTACE and H3 led to similar TL1A inhibition as H3 alone and was much less potent than A2 (Figure S4). These results highlight the importance of fusion of the two domains and are in accordance with the lack of pTACE ability to inhibit TL1A induced apoptosis in the TF-1 cells (Figure S3).

**Figure 5:**
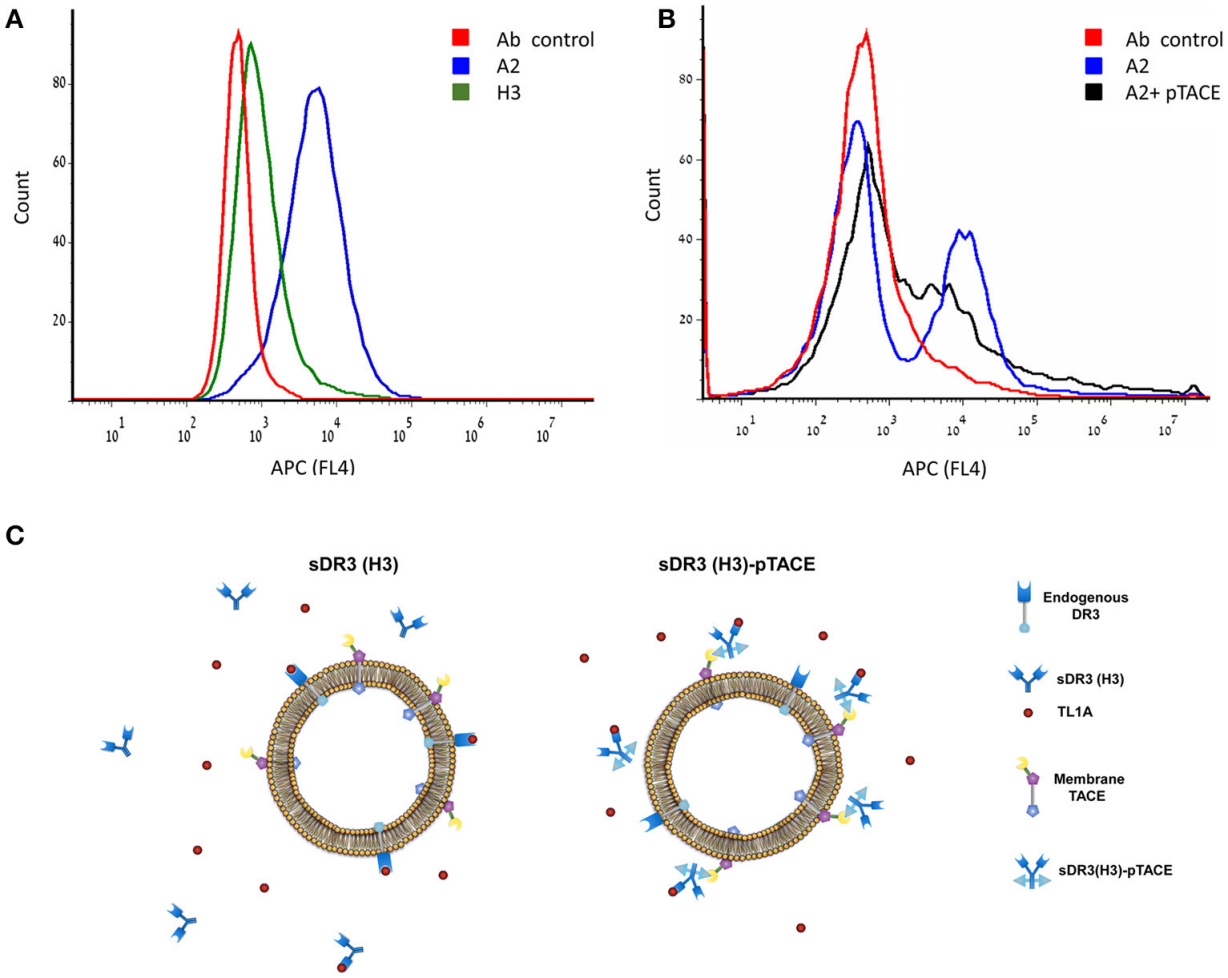
High binding of the A2 bi-specific inhibitor to TF-1 cells through the pTACE domain. **(A)** Flow cytometry histogram analysis of gated TF-1 cell population incubated with A2 (blue), H3 (green) and antibody control (without the addition of the A2 or H3, red). Binding to the cell membrane was analyzed following incubation with allophycocyanin (APC) fluorescent anti-human Fc. **(B)** A2 binds cells through the pTACE domain. Shown is a competition experiment where 100 nM A2 was incubated with TF-1 cells in the presence or absence of 15 μM pTACE inhibitor that lacks an Fc region. A decrease in cell labeled population is observed in the presence of pTACE suggesting that A2 binds cells through endogenous membrane TACE. **(C)** A model for TL1A inhibition by the mono-specific soluble DR3 (H3) and the A2 bi-specific DR3 (H3)-pTACE. Left, binding of H3 to TL1A leads to depletion of free TL1A due to a competition with the endogenous DR3 membrane receptor. Right, the bi-specific H3-pTACE is bound to cell surface TACE located on the cell membrane leading to a high local concentration of the inhibitor on the cell membrane. This cell targeting leads to increased potency of inhibition of TL1A induced apoptosis in TF-1 cells by up to ~80-fold (Figure 4).

An alternative mechanism that can explain the increased potency of A2 is by binding of the inhibitor to the cell membrane. A2 binding to cells will increase its local concentration on the cell surface. To test this hypothesis, we examined the binding of A2 and H3 to the TF-1 cell membrane following cell incubation. Binding was analyzed by cell labeling with allophycocyanin (APC) fluorescent anti-human Fc. Flow cytometry analysis of the labeled cells showed that while H3 binds very weakly to the TF-1 cells, A2 binds cells very efficiently providing direct evidence for the targeting of A2 to the cell membrane (Figure 5A). To show that A2 binds cells through its pTACE domain, we performed a competition experiment where we incubated TF-1 cells with A2 and pTACE domain that lacks an Fc region. The resulting cell population was labeled with APC-conjugated anti-Fc antibody and analyzed by flow cytometry. As expected, we observed a significant decrease in the mean cell fluorescence suggesting that pTACE domain can compete with A2 for binding to the cells (Figure 5B and Figure S5).

Overall, our results support a model in which the increased potency of A2 in inhibiting TL1A induced TF-1 apoptosis is due to increased local concentration of the bi-specific inhibitor on the cell membrane (Figure 5C). Since TL1A signals through binding to the endogenous DR3 cell surface receptor, enhanced local concentration of A2 on the cell membrane can increase the potency of TL1A inhibition.

## Discussion

Here, we developed a bi-specific inhibitor for targeting the TL1A pro-inflammatory cytokine and TACE catalytic activity. We found that this bi-specific inhibitor maintains the biochemical activity of each of the inhibitory domains and exhibits strong synergism between the domains in inhibiting TL1A induced apoptosis or IFN-γ secretion in different cell types. This synergism was associated with cell surface binding of the inhibitor through the pTACE domain leading to high local concentration of the inhibitor on the TF1 cell membrane (Figure 5C). Targeting soluble pro-inflammatory cytokines and membrane bound metalloprotease has high potential for increasing their inhibitory potency in a cell restricted manner. Several metalloproteases were previously shown to express under inflammatory conditions and were shown to be associated with disease progression (e.g., MMP-1, MMP-8, MMP-9, MMP-10, MMP-12, MMP-13, and ADAM-8; Shimshoni et al., 2015). Thus, our strategy can provide a general approach for targeting cells at sites of inflammation while inhibiting immunological signals that drive this process. Recently, TNF-α inhibitor fused to F4/80 scFv domain was generated for inhibiting TNF-α on macrophages obtaining a cell-type restricted TNF-α inhibition (Efimov et al., 2016). This bi-specific inhibitor proved to be much more effective *in vivo* than a systemic TNFα blockade. In our study the pTACE domain of the bi-specific inhibitor is utilized not only for the inhibition of TACE but also for cell targeting. Overall, our inhibitor exhibits three different functions: specific cell surface binding through pTACE, TACE and TL1A inhibition. Previously, high level of TACE mRNA expression was shown to be ubiquitous and found in many cell types (Scheller et al., 2011; Rose-John, 2013). However, active TACE was found at sites of inflammation and cancer suggesting the applicability of targeting cells that play a pathogenic role in these diseases (Scheller et al., 2011; Rose-John, 2013).

Another advantage of using the TL1A and pTACE domains fused to the Fc region of IgG is their small size and independent function enabling their natural bivalent assembly through Fc dimerization (Figure 1). This is in contrast to bi-specific antibodies that require complex assembly of the chains through the “knobs” and “holes” approach or other specific assembly approaches to prevent the generation of mono-specific antibodies (Todorovska et al., 2001; Spiess et al., 2013).

In summary, bi-specific inhibitors hold great promise for the therapeutics of multifactorial diseases such as autoimmune diseases. In recent years bi-specific antibodies were generated for targeting two disease promoting factors by the complex assembly of heavy and light chains. Our small inhibitory domains that readily assemble through the Fc domain allowed us to generate a potent bi-specific inhibitor for the inhibition of TL1A cytokine and TACE metalloprotease with potentially long serum half-life. TACE and TL1A were previously shown to promote IBD through the release of TNF-α (TACE) and immune cell differentiation (TL1A). Thus, the simultaneous inhibition of these factors can be highly beneficial in preventing IBD disease progression through blocking two complementary pro-inflammatory pathways. We believe that the synergism between the domains, shown in this study, holds high potential for the inhibition of IBD in animal disease models and possibly in human patients. The observation that IBD cells express high levels of active TACE on the cell membrane further supports this notion. Our inhibitor paves the way for the generation of many other bi-specific inhibitors targeting different metalloproteases including MMPs and inflammatory cytokines for fighting autoimmune inflammatory diseases, including IBD, through multiple complementary pathways.

## Author contributions

TW, IL, MZ, IS, and AA: designed research; TW, IL, and MZ: performed research; TW, IL, MZ, IS, and AA: analyzed data; and IL and AA: wrote the paper.

### Conflict of interest statement

The authors declare that the research was conducted in the absence of any commercial or financial relationships that could be construed as a potential conflict of interest.

## Abbreviations

IBD: Inflammatory bowel disease
TL1A: TNF-like ligand 1A
TACE: tumor necrosis factor-α-converting enzyme
ECM: extracellular matrix.
