# Supplementary material for: Increased Potency of a Bi-specific TL1A-ADAM17 (TACE) Inhibitor by Cell Surface Targeting
Source: Front Mol Biosci. 2017 Aug 22;4:61. doi: 10.3389/fmolb.2017.00061 (PMC5572276; doi:10.3389/fmolb.2017.00061)
Supplement: Supplementary file 1 [file Image1.PDF]

Supplementary information

Increased potency of a bi-specific TL1A-ADAM17 (TACE) inhibitor by cell surface targeting

Tomer Weizman[1,2,4], Itay Levin[1,2,4], Marianna Zaretsky[1,2], Irit Sagi[3] and Amir Aharoni[1,2*]

[1]Department of Life Sciences and [2]the National Institute for Biotechnology in the Negev, Ben-Gurion University of the Negev, Be'er Sheva 84105, Israel. [3]Department of Biological Regulation, Weizmann Institute of Science, Rehovot, Israel. [4]These authors contributed equally to this work.

[*] **Correspondence**: Amir Aharoni, Department of Life Sciences, Ben-Gurion University of the Negev, Be'er Sheva 84105, Israel

E-mail: aaharoni@bgu.ac.il

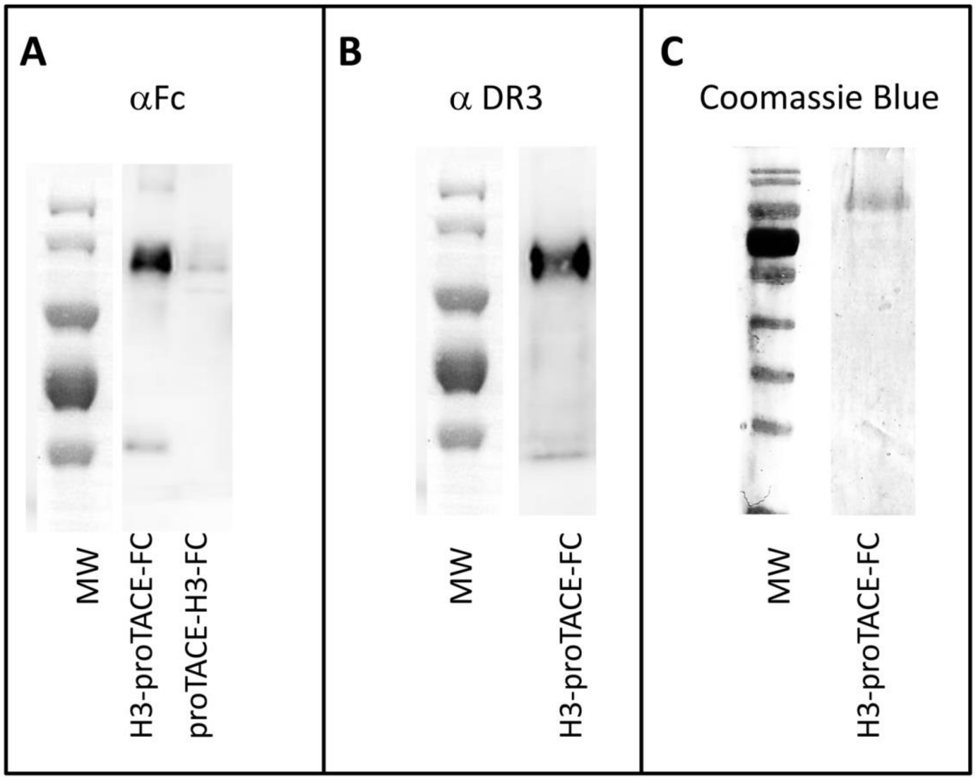

Figure S1: Analysis of the expression of the bispecific proTACE-H3-Fc (A1) and H3-proTACE-Fc (A2) constructs. (A) Western blot analysis of the expression of A1 and A2 constructs using anti-Fc antibody showing that A2 is expressed at a much higher levels. (B) Western blot analysis of the A2 construct using anti-DR3 polyclonal antibody. (C) Coomassie blue analysis of A2 following N-NTA purification.

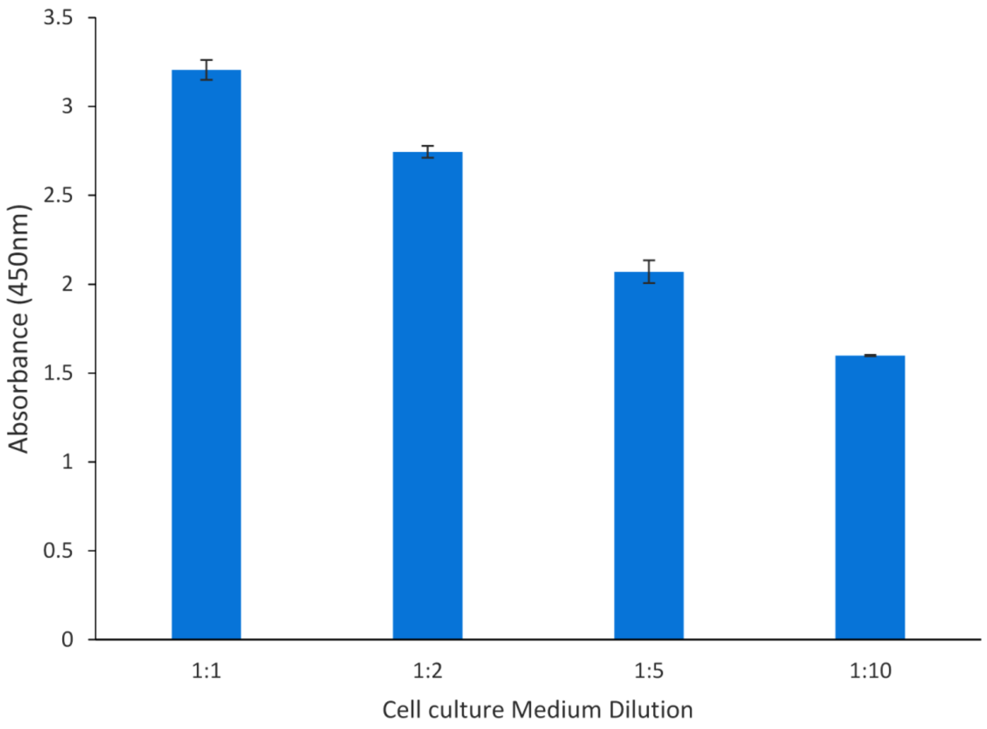

Figure S2: ELISA for the analysis of the bi-specific A2 binding to immobilized TL1A following expression in HEK293F mammalian cell line. Media containing the secreted A2 protein was directly applied to plates that were pre-coated with TL1A. The level of A2 binding to TL1A was determined using polyclonal anti-DR3 antibodies followed by secondary HRP linked antibody (see Material and Methods for detailed description).

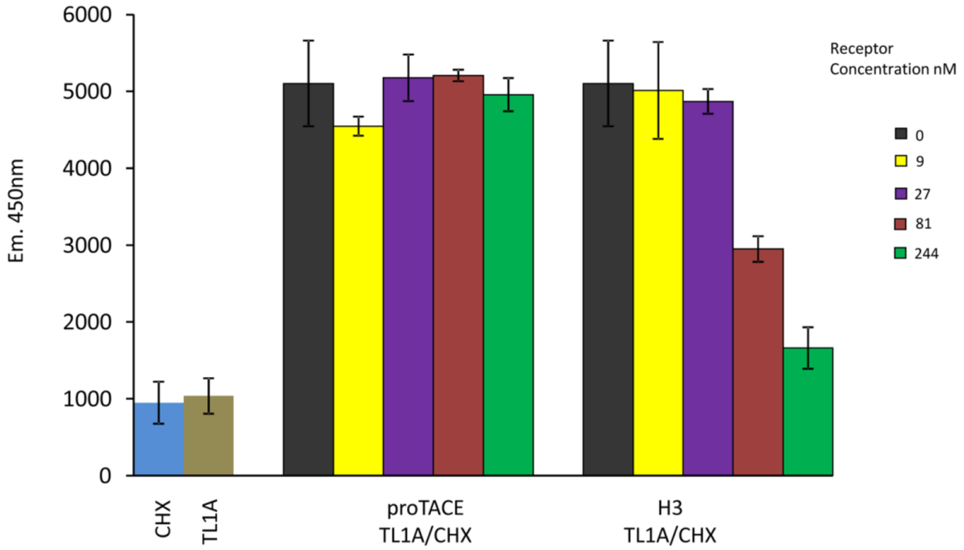

Figure S3: TL1A-induced apoptosis in TF-1 cells is not inhibited by pTACE. Cells were inoculated for six hours with 8 μg/ml cyclohexamide (CHX) and 75 ng/ml TL1A and the indicated concentration of pTACE. Following six hours of incubation, lysis buffer containing the caspase-3 fluorescent substrate DEVD-AMC was added and enzyme activity was monitored for 10 minutes. The data represented is the average of three independent repeats of each experiment and the error bars represent the standard deviation from the mean.

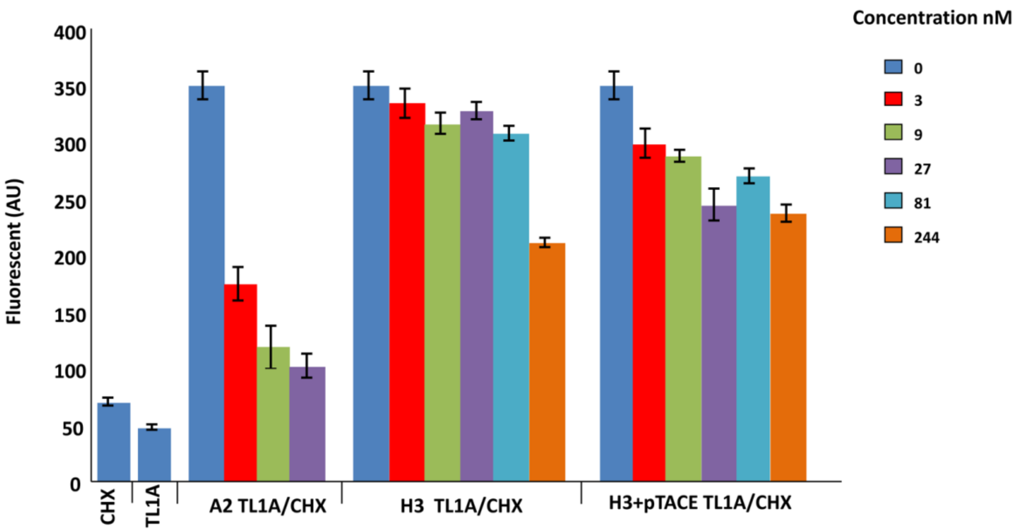

Figure S4: TL1A-induced apoptosis in TF-1 cells is weakly inhibited by the combination of H3 and pTACE. Cells were inoculated for six hours with 8 μg/ml cyclohexamide (CHX) and 75 ng/ml TL1A and the indicated concentration of A2, H3 or H3 and pTACE. Following six hours of incubation, lysis buffer containing the caspase-3 fluorescent substrate DEVD-AMC was added and enzyme activity was monitored for 10 minutes. The data represented is the average of three independent repeats of each experiment and the error bars represent the standard deviation from the mean.

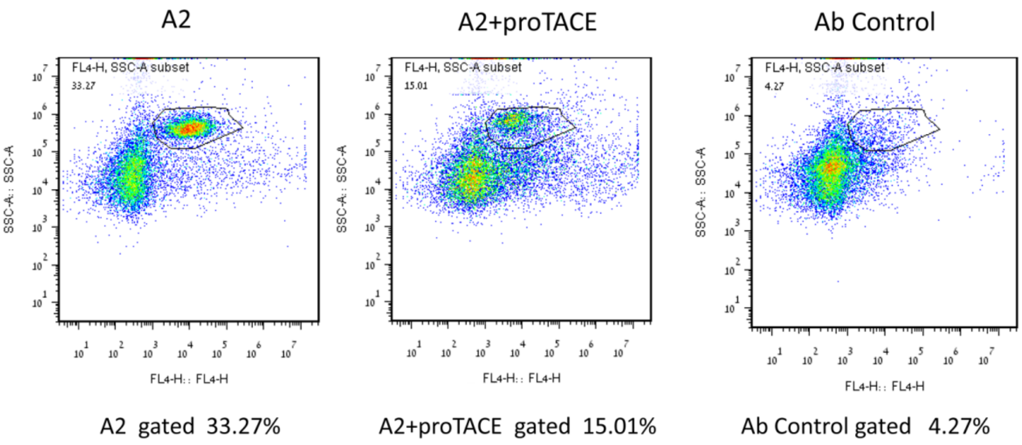

Figure S5: Dot plot analysis of TF-1 cell population incubated with A2 (left), A2+pTACE (middle) and antibody control (without the addition of the A2 or H3, right). A decreased in the gated population of the A2+pTACE labeled cells from 33.3% to 15% indicates that pTACE compete with the A2 for TF-1 cell binding.
